# CPAP Effect on Cardiopulmonary Exercise Testing Performance in Patients with Moderate-Severe OSA and Cardiometabolic Comorbidities

**DOI:** 10.3390/medicina56020080

**Published:** 2020-02-15

**Authors:** Ioana Mădălina Zota, Cristian Stătescu, Radu Andy Sascău, Mihai Roca, Radu Sebastian Gavril, Teodor Flaviu Vasilcu, Daniela Boișteanu, Alexandra Maștaleru, Alexandra Jitaru, Maria Magdalena Leon Constantin, Florin Mitu

**Affiliations:** 1 Department of Medical Specialties (I), Faculty of Medicine, Grigore T. Popa—University of Medicine and Pharmacy, 700115 Iași, Romania; madalina.chiorescu@gmail.com (I.M.Z.); cstatescu@gmail.com (C.S.); radu.sascau@gmail.com (R.A.S.); rgavril87@yahoo.com (R.S.G.); teodor.vasilcu@gmail.com (T.F.V.); alexandra.mastaleru@gmail.com (A.M.); alexandrajitaru@gmail.com (A.J.); leon_mariamagdalena@yahoo.com (M.M.L.C.); mitu.florin@yahoo.com (F.M.); 2 Department of Medical Specialties (III), Faculty of Medicine, Grigore T. Popa—University of Medicine and Pharmacy, 700115 Iași, Romania; boisteanu@yahoo.com

**Keywords:** obstructive sleep apnea, continuous positive airway treatment, cardiopulmonary exercise testing, functional capacity, cardiovascular rehabilitation

## Abstract

*Background and Objectives:* Obstructive sleep apnea (OSA) is associated with daytime somnolence, cognitive impairment and high cardiovascular morbidity and mortality. Obesity, associated cardiovascular comorbidities, accelerated erythropoiesis and muscular mitochondrial energetic dysfunctions negatively influence exercise tolerance in moderate-severe OSA patients. The cardiopulmonary exercise testing (CPET) offers an integrated assessment of the individual’s aerobic capacity and helps distinguish the main causes of exercise limitation. The purpose of this study is to evaluate the aerobic capacity of OSA patients, before and after short-term continuous positive airway pressure (CPAP). *Materials and Methods:* Our prospective study included 64 patients with newly diagnosed moderate-severe OSA (apnea hypopnea index (AHI) 39.96 ± 19.04 events/h) who underwent CPET before and after CPAP. Thirteen patients were unable to tolerate CPAP or were lost during follow-up. Results: 49.29% of our patients exhibited a moderate or severe decrease in functional capacity (Weber C or D). CPET performance was influenced by gender but not by apnea severity. Eight weeks of CPAP induced significant improvements in maximal exercise load (Δ = 14.23 W, *p* = 0.0004), maximum oxygen uptake (Δ = 203.87 mL/min, *p* = 0.004), anaerobic threshold (Δ = 316.4 mL/min, *p* = 0.001), minute ventilation (Δ = 5.1 L/min, *p* = 0.01) and peak oxygen pulse (Δ = 2.46, *p* = 0.007) as well as a decrease in basal metabolic rate (BMR) (Δ = −8.3 kCal/24 h, *p* = 0.04) and average Epworth score (Δ = −4.58 points, *p* < 0.000001). *Conclusions:* Patients with moderate-severe OSA have mediocre functional capacity. Apnea severity (AHI) was correlated with basal metabolic rate, resting heart rate and percent predicted maximum effort but not with anaerobic threshold or maximum oxygen uptake. Although CPET performance was similar in the two apnea severity subgroups, short-term CPAP therapy significantly improved most CPET parameters, suggesting that OSA per se has a negative influence on effort capacity.

## 1. Introduction

Repetitive nocturnal upper airway collapse, with subsequent hypoxic episodes and microawakenings, is the hallmark of obstructive sleep apnea (OSA) [1]. While chronic sleep fragmentation leads to excessive daytime somnolence and cognitive impairment [2], hypoxia is associated with autonomic and hormonal imbalance, endothelial dysfunction and oxidative stress [3], explaining the high cardiovascular morbidity and mortality described among OSA patients [1,3].

In-hospital polysomnography is the diagnostic standard for OSA, with cardio-respiratory polygraphy considered an acceptable alternative [4,5,6]. According to the apnea–hypopnea index (AHI), defined as the number of apneic or hypopneic episodes per hour of sleep, OSA is classified as mild, moderate or severe [7]. Daytime sleepiness is the main symptom in OSA, a subjective parameter that can be objectively assessed using the Epworth questionnaire.

Treatment is recommended in all cases of moderate-severe OSA (AHI ≥ 15 events/h), as well as in patients with mild OSA who associate symptoms or cerebrovascular comorbidities [8]. Current therapy options include continuous positive airway pressure (CPAP), mandibular advancement devices, maxillo-facial surgery and nocturnal hypoglossal nerve stimulation [9,10]. Although CPAP remains the gold-standard treatment option for moderate-severe OSA, its use is limited by poor treatment adherence, especially among children.

Obesity and weight-related lung-function abnormalities (decreased functional residual capacity and expiratory reserve volume, impaired respiratory system compliance) are highly prevalent among OSA patients [10]. Associated cardiovascular comorbidities (hypertension, heart failure, pulmonary hypertension), hypoxia-induced erythropoiesis [11] with subsequent hematological alterations and muscular mitochondrial dysfunctions also contribute to a decreased exercise tolerance [10,12]. The cardiopulmonary exercise testing (CPET) provides an integrative assessment of the cardiopulmonary, muscular, neuropsychological and hematopoietic systems, which directly impact the individual’s functional capacity [13]. CPET is a valuable cardiovascular instrument for risk stratification and prognosis assessment, helping to establish a personalized exercise training program for OSA patients. Current literature [14] offers conflicting results regarding CPET results in OSA patients and the role of CPAP in improving exercise performance. As such, the purpose of this study is to evaluate the impact of short-term (8 weeks) CPAP therapy on exercise capacity of patients with moderate-severe OSA and cardiometabolic comorbidities.

## 2. Materials and Methods

We performed a prospective study that included newly diagnosed patients with moderate-severe OSA (prior to the initiation of CPAP therapy), admitted in our local cardiovascular rehabilitation clinic between October 2017 and December 2018. OSA diagnosis was made by ambulatory or in-hospital six-channel cardio-respiratory polygraphy, using either a Philips Respironics Alice Night One or a DeVilbiss Porti 7 device. The recordings were manually scored by a trained physician, according to the American Academy of Sleep Medicine (AASM) standards. Patients with an apnea–hypopnea index (AHI) of 15–30 and >30 were considered to have moderate and severe OSA, respectively. A Philips Respironics DreamStation Auto CPAP or a Resmed Airsense 10 Autoset were used for CPAP effective pressure autotitration in the sleep laboratory.

All patients signed a written informed consent for inclusion. The study was conducted in accordance with the Declaration of Helsinki, and the protocol was approved by the Ethics Committee of the ”Grigore T. Popa” University of Medicine and Pharmacy in Iași (ethical approval code 1183). All subjects underwent physical examination, lipid profile, cardiopulmonary exercise testing and were asked to complete the Epworth questionnaire, before and after 2 months of CPAP therapy. Obesity was defined as a body mass index (BMI ≥ 30 kg/m^2^). High blood pressure (HBP) was defined as current BP lowering treatment, prior diagnosis of HBP or resting BP values greater than 140 and 90 mmHg for systolic and diastolic BP, respectively. Dyslipidemia was defined as total cholesterol ≥ 200 mg/dL and/or triglycerides ≥ 150 mg/dL. Ischemic heart disease was defined as history of myocardial infarction or prior angiographically documented significant coronary artery stenosis. According to the results of the Epworth questionnaire, daytime sleepiness was categorized as normal, mild, moderate and severe (0–10 points, 11–12 points, 13–15 points and 16–24 points, respectively). Functional capacity was assessed according to peak oxygen uptake (VO2), using the Weber classification, as follows: Weber A (little or no impairment): >20 mL/kg/min, Weber B (mild to moderate impairment): 16–20 mL/kg/min, Weber C (moderate to severe impairment): 10–16 mL/kg/min and Weber D (severe impairment) < 10 mL/kg/min. 

CPET was performed under the direction of a certified pulmonologist on the Piston PRE-201 ergospirometer. This started with a 2 min resting period followed by 1 min warm up pedaling against no resistance and an incremental test protocol of 10 W/min. The CPET was performed under continuous heart rate (HR), ECG (electrocardiographic) and pulse oximetry (SpO2) monitoring. BP was recorded every 2 min. Indications for exercise termination included extreme fatigue, myocardial ischemia, complex ventricular premature beats, grade 2 or grade 3 atrio-ventricular block, a sudden drop in BP levels by more than 20 mmHg, increased BP (systolic blood pressure (SBP) > 220 mmHg, diastolic blood pressure (DBP) > 120 mmHg), SpO2 < 80%, confusion, dizziness and sudden pallor.

Statistical analysis was performed in SPSS v. 20.0, using chi-square and student’s t-test for comparisons between groups. A potential relationship between variables was evaluated using Pearson’s correlation coefficient. The ANCOVA test was used for BMI-adjusted comparison of CPET performance before and after CPAP use. Descriptive data were expressed as means ± SD (standard deviation) or percentages, as appropriate. A *p* value < 0.05 was considered statistically significant.

## 3. Results

**Figure 1:**
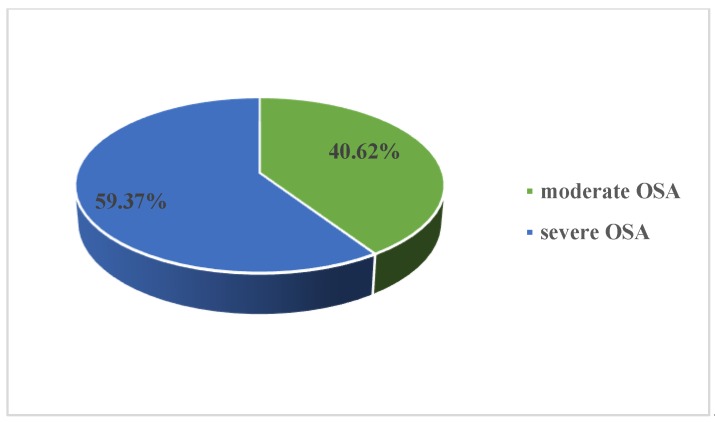
Prevalence of moderate and severe obstructive sleep apnea (OSA) in our study group.

**Figure 2:**
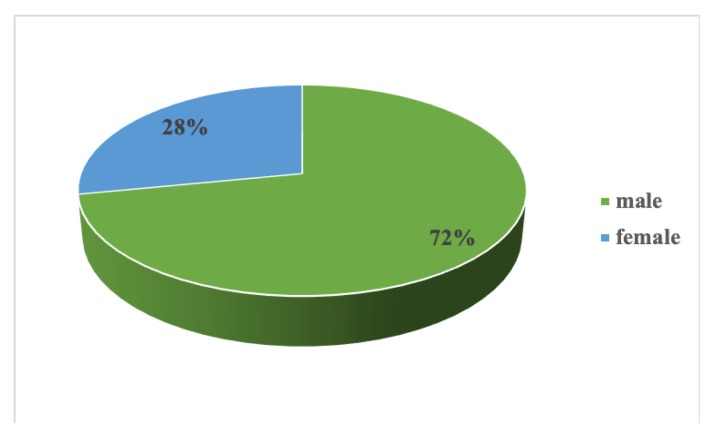
Gender distribution in our study group.

**Figure 3:**
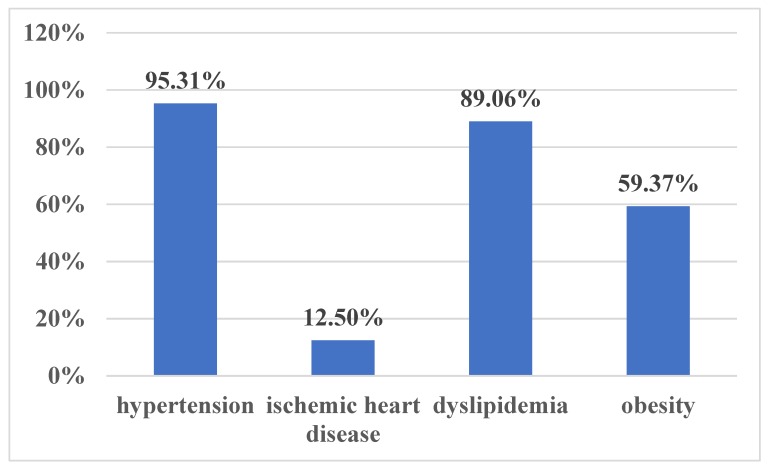
Prevalence of cardio-metabolic comorbidities in our study group.

Our initial study group included 64 patients aged 36–79 years old (57.53 ± 8.74 years old), mean BMI 34.04 ± 5.30 kg/m^2^, with newly diagnosed OSA (AHI 39.96 ± 19.04 events/h, desaturation index 38.67 ± 19.67 events/h, average nocturnal SpO2 91.63% ± 3.64%, CPAP pressure 11.27 ± 2.43 cmH20). Almost two-thirds of our study group presented severe OSA (59.37%) (Figure 1). Male sex was predominant in our study group, with a M/F ratio of 2.55 (Figure 2). Cardiometabolic comorbidities (particularly hypertension) were highly prevalent among our patients (Figure 3).

**Figure 4:**
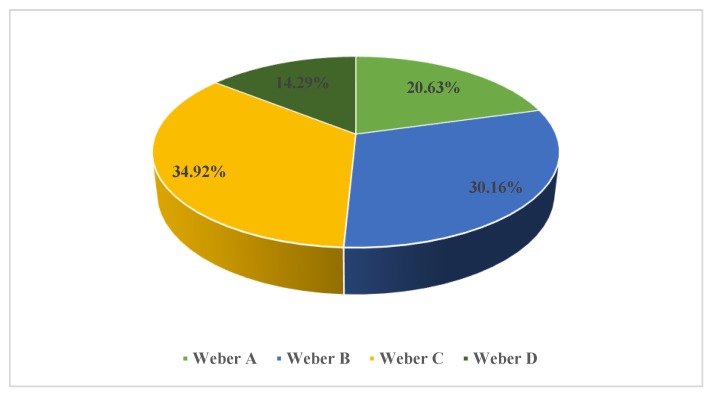
Functional capacity in our study group according to the Weber classification.

Of our patients, 49.21% exhibited a moderate or severe decrease in functional capacity, according to the Weber classification (Weber C or D) (Figure 4). Only one in five patients with moderate-severe OSA had a normal functional capacity (Weber A). We found no significant differences regarding average AHI values between the four functional capacity subgroups (Weber A to D) (*p* > 0,05).

Apart from maximal instantaneous forced expiratory flow (MEF)25% that was higher in the severe OSA subgroup, we did not find any statistically significant differences regarding spirometry results between patients with moderate and severe OSA (Table 1).

CPET performance was influenced by gender but not by apnea severity (Table 2 and Table 3).

Except for baseline SBP, CPET parameters did not significantly differ between the two apnea severity subgroups (Table 2). Basal metabolic rate (BMR) and minute ventilation (VE) max were significantly higher among males (∆ = 366 kCal/24 h and ∆ = 8.35 L/min, respectively). Although males achieved a higher average peak workload (∆ = 34.07 W), % predicted workload and % predicted VO2 max were significantly higher in the female subgroup (∆ = 13.33% and ∆ = 20.24%, respectively).

**Figure 5:**
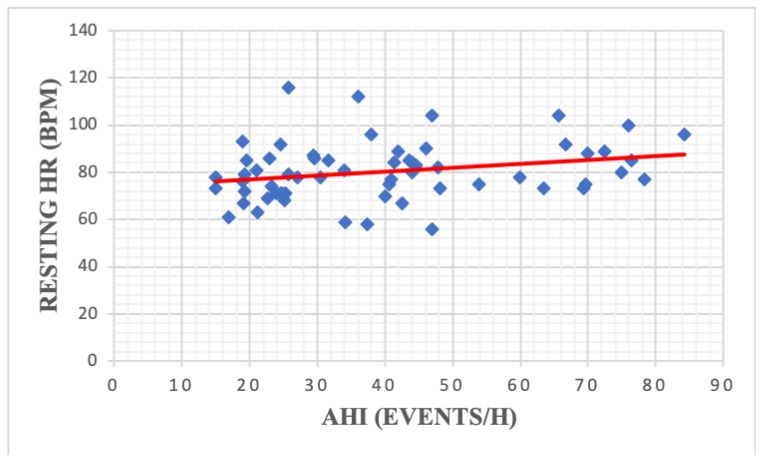
Correlation between apnea severity and resting heart rate among patients with moderate-severe OSA (r = 0.25, *p* = 0.04). HR—heart rate; AHI—apnea hypopnea index; OSA—obstructive sleep apnea.

**Figure 6:**
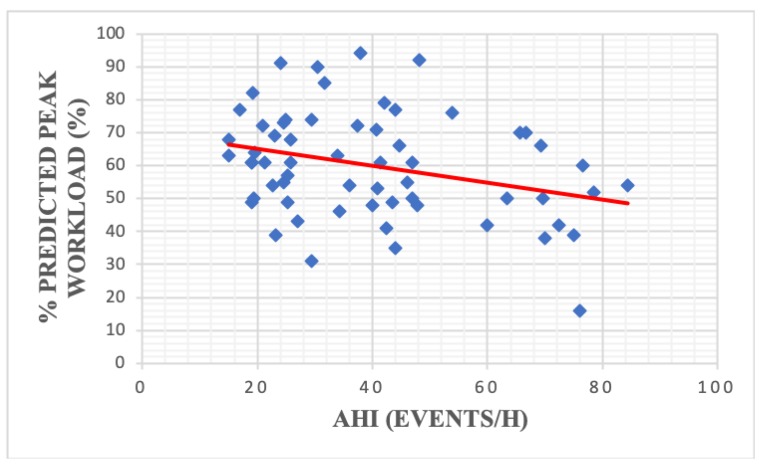
Correlation between apnea severity and % predicted peak workload among patients with moderate-severe OSA (r = −0.30, *p* = 0.01). AHI—apnea hypopnea index; OSA—obstructive sleep apnea.

**Figure 7:**
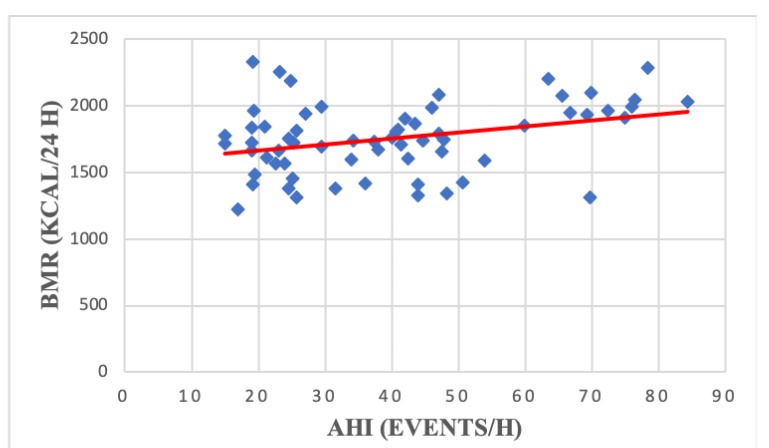
Correlation between apnea severity and BMR among patients with moderate-severe OSA (r = 0.33, *p* = 0.008). BMR—basal metabolic rate; AHI—apnea hypopnea index; OSA—obstructive sleep apnea.

Apnea severity was significantly correlated with resting HR (r = −0.30, *p* = 0,01) (Figure 5), % predicted workload (r = −0.30, *p* = 0.01) (Figure 6) and BMR (r = 0.33, *p* = 0.008) (Figure 7) (Table 4). We did not find any statistically significant correlations between AHI and the analyzed spirometry parameters (*p* > 0.05).

All subjects started appropriate continuous positive airway pressure therapy. Thirteen patients were unable to tolerate CPAP or were lost during follow-up. Fifty-one patients successfully completed the CPET and the Epworth questionnaires before and after 2 months of CPAP. 

The average Epworth score in our study group was 8.11 ± 5.23 points. Average CPAP use was 241.67 (±128.38) minutes/night. Only 51.16% of our patients used the device as recommended—at least 4 h/night. CPAP use did not significantly impact basal blood pressure values (SBP ∆ = −4.58 mmHg, *p* = 0.13; DBP ∆ = −1.52 mmHg, *p* = 0.35) and was not associated with statistically significant weight loss (∆ = −1.01 kg, *p* = 0.57).

**Figure 8:**
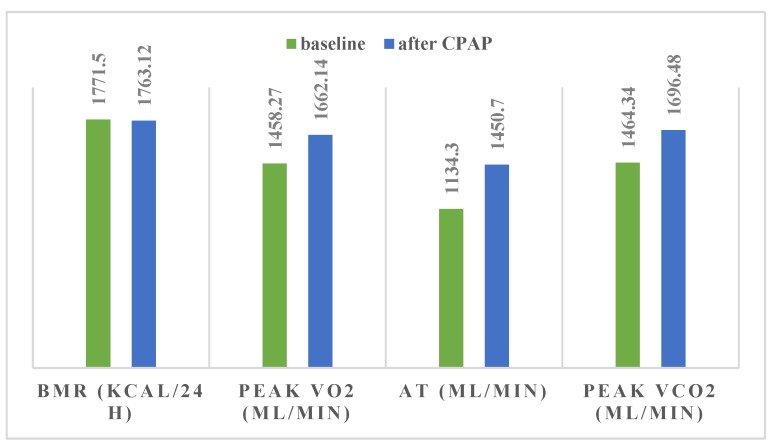
CPAP induced changes in BMR (Δ = −8.38 kCal/24 h, *p* = 0.04), peak VO2 (Δ = 203.87 mL/min, *p* = 0.004), AT (Δ = 316.4 mL/min, *p* = 0.001) and peak VCO2 max (Δ = 232.14 mL/min, *p* = 0.0006). BMR—basal metabolic rate; peak VO2—peak oxygen uptake; AT—anaerobic threshold; peak VCO2—peak CO2 output.

**Figure 9:**
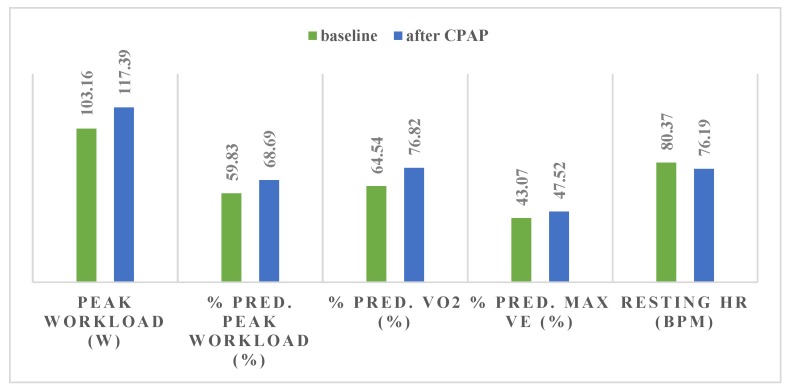
CPAP induced changes in peak workload (Δ = 14.23 W, *p* = 0.0004), % predicted peak workload (Δ = 8.86 %, *p* = 0.0001), % predicted peak VO2 (Δ = 12.28 %, *p* = 0.000005), % predicted VE max (Δ = 4.45 %, *p* = 0.01) and resting HR (Δ = −4.18 bpm, *p* = 0.05), in moderate-severe OSA patients. CPAP—continuous positive airway pressure; OSA—obstructive sleep apnea; VO2—peak oxygen uptake; VE—minute ventilation; HR—heart rate.

After 2 months of CPAP our study group exhibited significant improvements in maximal exercise load (Δ = 14.23 W, *p* = 0.0004), VO2 max (Δ = 203.87 mL/min, *p* = 0.004), anaerobic threshold (AT) (Δ = 316.4 mL/min, *p* = 0.001) and VE max (Δ = 5.1 L/min, *p* = 0.01) (Table 5, Figure 8 and Figure 9). Maximal exercise load and VO2 max improvement remained significant after adjustment for BMI (Table 5. *p* = 0.04 and *p* = 0.02, respectively). We also observed an increase in peak oxygen pulse (Δ = 2.46, *p* = 0.007) and VCO2 max (Δ = 232.14 mL/min, *p* = 0.0006), which remained significant after adjusting for BMI (Table 5, Figure 8 and Figure 9, *p* = 0.02 and *p* = 0.01, respectively). The Epworth score in our study group decreased by 4.58 points (*p* < 0.000001).

## 4. Discussion

Our study included 64 patients aged 57.53 ± 8.74 years old with newly diagnosed moderate-severe OSA. This value is slightly higher than other reports concerning average OSA age at diagnosis (40–50 years old) [15]. Female sex hormones increase genioglossus contractility and prevent upper airway collapsibility during sleep [16,17]. Furthermore, the distinctive distribution of adipose tissue among the two genders (with central obesity being more strongly associated with OSA) [18], as well as the higher pharyngeal resistance in men [19], explain why OSA is more prevalent among male patients. Despite the evident predominance of the male sex in our study group, our male/female ratio is slightly lower than in previous studies (2.55:1 vs. 3:1–5:1) [20].

Similar to other literature reports [21], the main reason for premature test halt was dyspnea accompanied by muscular exhaustion. Extreme fatigue in OSA patients can be explained by the presence of energetic mitochondrial dysfunctions especially in muscle cells [12]. An exaggerated SBP response (SBP > 250 mmHg) was the second reason for premature test halt. None of our patients presented arrhythmic events, confusion or a decrease in BP values during exercise.

Our moderate-severe OSA patients presented baseline mediocre CPET performance. Only 20.63% of our subjects had a baseline normal functional capacity according to the Weber classification, and most cases (34.92%) were classified as moderately impaired. In comparison, Przybyłowski et al. [22] reported an overall better CPET performance in 111 obese OSA patients (% predicted peak VO2 85.3 ± 17.8, peak VCO2 2800 ± 900 mL/min, VE max 91.2 ± 24.7, % predicted maximum HR 92.5 ± 10.3), despite a minimal difference in OSA severity between the two groups (average AHI 47.2 ± 23.1 vs. 39.96 ± 19.04 events/h). However, Przybyłowski’s study group included an unusually low percentage of hypertensives (29% vs. 95.31% in our study group), signaling that HBP could be an important confounding factor when analyzing CPET performance.

Previous reports regarding the impact of OSAS on cardiopulmonary exercise testing performance have conflicting results and included a limited number of patients [10,12,23,24,25]. Most studies that associated OSAS with an impaired exercise capacity (decreased exercise duration, workload, VO2, oxygen pulse, AT and/or VE max) were conducted on obese or overweight subjects [10,12,25]. Therefore, these reports could be biased by the known negative impact of obesity on exercise capacity, as shown by Rizzi et al. [26].

Consistent with this theory, another report [27] found that CPET performance is similar among normoponderal OSAS patients and controls, although it is worth mentioning that the analyzed group had a relatively low average AHI (15.4 ± 9.2) and included an unusually large proportion of females (63%).

Powell et al. [28] studied exercise performance among military personnel with and without moderate-severe OSAS. The lack of significant differences among the two subgroups could be explained by the low average age in the OSAS and control groups (40.7 and 39.4, respectively) but also by the higher grade of habitual physical activity (characteristic for this population subset) [29].

However, a recent meta-analysis [29] has shown that VO2 max is significantly lower in OSA subjects compared to controls (Δ = 2.7 mL/kg/min), the difference being of greater clinical impact among non-obese patients (Δ = 4.1 mL/kg/min).

Rizzi et al. [26] reported that male sex associated with diabetes negatively impacts VO2 max. Consistent with their results, our female subgroup obtained significantly higher percent predicted workload and percent-predicted maximum HR, suggesting a higher effort capacity.

Apnea severity was previously correlated with several CPET parameters including VO2 max [25], percent predicted peak VO2 [30] and BP rise during exercise [22]. However, our analysis only found a significant association between AHI resting HR, BMR and percent predicted workload. The high prevalence of cardio-metabolic comorbidities in our study group (especially obesity and hypertension) could explain the lack of statistically significant correlations between AHI and other CPET variables.

Previous studies [24,25,29] reported that OSAS patients have higher DBP values and decreased HR recovery compared to controls. When analyzing the two apnea severity subgroups, we observed significantly higher baseline and AT-SBP values (but no significant differences regarding HR response during exercise) in the severe OSA subgroup.

Literature reports regarding the impact of CPAP on VO2 max in OSAS patients have yielded inconsistent results. Different CPAP therapy lengths (1 week–8 months) were associated with significant VO2 max improvements [29,31,32,33]. However, in another study [34], VO2 max displayed a mild negative trend (22.52 ± 6.62 mL/min/kg to 21.32 ± 5.26 mL/min/kg; *p* = 0.111) in CPAP compliant patients and a borderline statistically significant decline in patients with suboptimal CPAP use (21.31 ± 5.66 mL/min/kg to 19.92 ± 5.40 mL/min/kg, *p* = 0.05).

Despite a mediocre CPAP adherence (241.67 min/night), our patients exhibited a significant improvement in percent predicted maximum workload, percent predicted VO2 max, AT and oxygen pulse. Improvements regarding maximal load, VO2 max, VCO2 max, %VE and peak O2 pulse remained significant even after adjusting for BMI. We observed no statistically significant gender-related differences regarding these changes. Quadri et al. [33] also studied the effect of 2 months of CPAP in a smaller group of moderate-severe OSAS patients and reported similar improvements in percent predicted maximum workload (9 vs. 8.86 W%) and percent predicted VO2 peak (9.7% vs. 12.28%) but a less marked increase regarding AT (99 vs. 316.4 mL/min). On the other hand, Tapan et al. [21] analyzed the benefit of 8 weeks of CPAP in patients with severe OSA and observed a greater improvement in maximum workload and VE (16.9 W and 10.3 L/min respectively) but a less important increase in percentage-predicted peak VO2 (7.6% vs. 12.28%).

Previous research [35] reported diurnal variations in spirometric indices in OSA patients (especially among males). The same study [35] observed significant associations between AHI, evening forced expiratory volume in one second (FEV1) and forced vital capacity (FVC) and demonstrated the important influence of BMI, hypertension, dyslipidemia and several cardiovascular drugs on the relationship between lung function and apnea severity. The fact that most of our patients presented cardio-metabolic comorbidities, and were under treatment with a statin, beta blocker or a renin-angiotensin-aldosterone axis inhibitor [35], could explain the lack of association between AHI and the analyzed spirometry parameters (*p* > 0.05).

The main limitations of our study are the lack of a control group and the high prevalence of cardio-metabolic comorbidities among our patients. Although obesity and hypertension are important confounders regarding the decrease in CPET performance described in OSAS patients, the presence of these comorbidities reflects the typical, everyday OSAS patient and, in our opinion, should not be excluded from analysis. Although baseline CPET results did not significantly differ between the two apnea severity subgroups, the fact that our 2 months of CPAP improved most CPET parameters in the absence of statistically significant weight loss (∆ = −1.01 kg, *p* = 0.57) or basal BP changes (SBP ∆ = −4.58 mmHg *p* = 0.13; DBP ∆ = −1.52 mmHg *p* = 0.35) suggests that OSAS per se impacts exercise capacity.

## 5. Conclusions

Moderate-severe OSA patients have a mediocre baseline CPET performance. AHI was correlated with some CPET parameters (BMR, % predicted effort, resting HR) but not with VO2 or AT. Two months of CPAP improved most CPET parameters (in the absence of statistically significant weight loss or basal BP changes) suggesting that OSAS per se negatively impacts effort capacity.

## Figures and Tables

**Table 1:** Spirometry results in patients with moderate-severe OSA.

	Moderate-Severe OSA		Moderate OSA		Severe OSA		*p*
	Average	SD	Average	SD	Average	SD	
FVC (L)	3.76	0.92	3.84	0.85	3.71	0.97	0.46
FVC% (%)	94.89	19.03	94.00	18.47	95.45	19.67	0.79
FEV1.0 (L)	3.05	0.68	3.12	0.66	3.00	0.69	0.54
FEV 1.0% (%)	91.61	19.96	88.00	19.39	93.90	20.30	0.31
FEV1.0/FVC	77.24	2.12	77.54	1.86	77.06	2.28	0.44
FEV1.0/FVC%	101.20	10.82	97.67	11.79	103.32	9.80	0.07
PEF (L/sec)	7.64	1.37	7.73	1.31	7.59	1.42	0.72
PEF% (%)	77.00	19.63	75.22	18.92	78.07	20.28	0.63
MEF 25 (L/sec)	1.59	0.39	1.65	0.37	1.55	0.41	0.43
MEF 25% (%)	69.90	23.39	61.17	24.04	75.33	21.64	0.04
MEF 50 (L/sec)	4.22	0.61	4.28	0.58	4.17	0.63	0.56
MEF 50% (%)	75.93	25.80	68.11	27.96	80.78	23.56	0.10
MEF 75 (L/sec)	6.74	1.16	6.80	1.12	6.69	1.20	0.75
MEF 75% (%)	76.44	20.75	72.89	21.67	78.65	20.22	0.36

FVC—forced vital capacity; FVC%—percent predicted forced vital capacity; FEV1—forced expiratory volume in one second; FEV1%—percent predicted forced expiratory volume in one second; PEF—peak expiratory flow; PEF%—percent predicted peak expiratory flow; MEF—maximal instantaneous forced expiratory flow; MEF%—percent predicted maximal instantaneous forced expiratory flow.

**Table 2:** Gender influence on cardiopulmonary exercise testing (CPET) parameters among patients with moderate-severe OSA.

	Male		Female		*p*
	Average	SD	Average	SD	
BMR (kCal/24 h)	1860.18	264.14	1494.06	187.26	<0.0000001
Maximal load (W)	114.85	33.54	80.78	23.41	0.0001
% predicted maximal load	56.74	16.04	70.07	15.49	0.004
VO2 max	1553.20	504.55	1301.67	352.11	0.07
% predicted VO2 max	61.59	21.73	81.83	15.56	0.0005
AT	1202.82	397.46	1083.07	288.10	0.33
Weight-indexed AT	11.82	3.79	11.83	3.15	0.99
VCO2 max	1434.52	460.30	1392.83	330.89	0.75
VE max (L/min)	48.64	12.88	40.29	8.79	0.02
Resting HR	79.20	12.27	83.80	15.85	0.21
Peak HR	117.02	19.34	123.73	18.52	0.25
% predicted peak HR	71.48	11.62	77.80	11.29	0.07
Peak O2 pulse	14.46	5.46	12.37	5.40	0.20
Weight-indexed O2 pulse	0.14	0.06	0.14	0.07	0.97
Baseline SBP	124.72	16.05	127.87	16.12	0.51
Baseline DBP	78.46	9.83	79.27	13.29	0.78
Peak SBP	183.16	28.01	185.33	18.85	0.80
Peak DBP	98.64	17.61	102.53	9.52	0.46

CPET—cardiopulmonary stress test; OSA—obstructive sleep apnea; BMR—basal metabolic rate; VO2—peak oxygen uptake; AT—anaerobic threshold; VCO2—peak CO2 output; VE—minute ventilation; HR—heart rate; SBP—systolic blood pressure; DBP—diastolic blood pressure.

**Table 3:** Differences regarding CPET parameters between moderate and severe OSA subgroups.

	Moderate-Severe OSA		Moderate OSA		Severe OSA		*p*
	Average	SD	Average	SD	Average	SD	
BMR (kCal/24 h)	1755.57	264.14	1726.27	276.57	1776.16	256.87	0.46
Maximal load (W)	105.27	33.54	111.08	34.20	101.29	32.94	0.25
% predicted maximal load	60.02	16.04	61.84	13.81	58.75	17.50	0.46
VO2 max	1482.45	504.55	1464.31	473.61	1494.87	530.57	0.81
% predicted VO2 max	67.28	21.73	67.27	21.98	67.29	21.86	0.99
AT	1168.92	397.46	1109.32	367.95	1211.23	417.85	0.36
Weight-indexed AT	11.83	3.79	11.59	4.24	12.00	3.49	0.70
VCO2 max	1422.80	460.30	1421.54	476.78	1423.66	455.16	0.98
VE max (L/min)	46.58	12.88	47.12	9.21	46.21	15.03	0.78
%VE	43.05	12.51	41.89	15.86	44.05	10.40	0.57
Resting HR	80.33	12.27	77.88	11.59	82.03	12.59	0.19
Peak HR	118.67	19.34	121.84	18.81	116.47	19.66	0.29
% predicted maximum HR	73.03	11.62	74.68	11.70	71.89	11.59	0.36
Peak O2 pulse	13.95	5.46	14.00	5.34	13.91	5.62	0.95
Weight-indexed O2 pulse	0.14	0.06	0.15	0.06	0.14	0.06	0.42
Baseline SBP	125.49	16.05	120.04	11.64	129.28	17.69	0.02
Baseline DBP	78.66	9.83	76.88	7.32	79.89	11.18	0.24
Peak SBP	183.70	28.01	175.50	26.32	189.17	28.12	0.06
Peak DBP	99.62	17.61	97.79	13.01	100.83	20.20	0.51

CPET—cardiopulmonary stress test; OSA—obstructive sleep apnea; BMR—basal metabolic rate; VO2—peak oxygen uptake; AT—anaerobic threshold; VCO2—peak CO2 output; VE—minute ventilation; HR—heart rate; SBP—systolic blood pressure; DBP—diastolic blood pressure.

**Table 4:** Correlations between AHI and CPET results among patients with moderate-severe OSA.

	r	*p*		r	*p*
BMR (kCal/24 h)	0.33	0.008	VCO2 max	0.10	0.42
Maximal load (W)	−0.07	0.55	VE max (L/min)	0.05	0.72
% predicted maximal load	−0.30	0.01	Resting HR	0.25	0.04
VO2 max	0.02	0.88	Peak HR	−0.12	0.33
% predicted VO2 max	−0.20	0.10	% predicted peak HR	−0.21	0.09
AT	0.15	0.28	Peak O2 pulse	−0.05	0.67
Weight-indexed AT	−0.02	0.85	Weight-indexed O2 pulse	−0.19	0.13

AHI—apnea hypopnea index; CPET—cardiopulmonary stress test; OSA—obstructive sleep apnea; BMR—basal metabolic rate; VO2—peak oxygen uptake; AT—anaerobic threshold; VCO2—peak CO2 output; VE—minute ventilation; HR—heart rate.

**Table 5:** CPAP impact on CPET parameters in moderate-severe OSA patients.

	Baseline		After CPAP		*p* *	*p* **
	Average	SD	Average	SD		
BMR (kCal/24 h)	1771.50	281.49	1763.12	273.79	0.04	0.78
Maximal load (W)	103.16	34.21	117.39	36.17	0.0004	0.04
% predicted maximal load	59.83	16.48	68.69	14.35	0.0001	0.01
VO2 max	1458.27	435.29	1662.14	454.50	0.004	0.02
% predicted VO2 max	64.54	17.49	76.82	18.47	0.000005	0.001
AT	1134.30	419.42	1450.70	450.54	0.001	0.08
Weight-indexed AT	11.41	4.07	14.62	4.66	0.001	0.07
VCO2 max	1464.34	383.13	1696.48	465.62	0.0006	0.01
VE max (L/min)	46.46	13.57	51.56	14.23	0.016	0.09
%VE	43.07	12.69	47.52	11.48	0.04	0.04
Resting HR	80.37	13.15	76.19	14.46	0.05	-
Peak HR	118.22	20.06	121.67	23.94	0.28	-
% predicted maximum HR	72.59	11.84	73.84	11.96	0.42	-
Peak O2 pulse	13.59	4.14	16.05	5.83	0.007	0.02
Weight-indexed O2 pulse	0.14	0.05	0.16	0.06	0.01	0.26
Baseline SBP	126.77	17.63	122.19	15.93	0.13	-
Baseline DBP	78.94	10.29	77.42	8.77	0.35	-
Peak SBP	184.62	29.35	185.35	23.27	0.85	-
Peak DBP	101.52	13.74	98.33	10.40	0.11	-

CPAP—continuous positive airway pressure; CPET—cardiopulmonary stress test; OSA—obstructive sleep apnea; BMR—basal metabolic rate; VO2—peak oxygen uptake; AT—anaerobic threshold; VCO2—peak CO2 output; VE—minute ventilation; HR—heart rate; SBP—systolic blood pressure; DBP—diastolic blood pressure; *p* *—statistical significance for non-adjusted student’s t-test; *p* **—statistical significance for BMI-adjusted results of ANCOVA test.
